# Toric Intraocular Lens Implantation in the Correction of Moderate-To-High Corneal Astigmatism in Cataract Patients: Clinical Efficacy and Safety

**DOI:** 10.1155/2021/5960328

**Published:** 2021-01-20

**Authors:** Xiaodi Qiu, Yumeng Shi, Xiaoyan Han, Zhixiang Hua, Yi Lu, Jin Yang

**Affiliations:** ^1^Eye Institute, Eye and Ear, Nose, and Throat, Hospital of Fudan University, 83 Fenyang Road, Shanghai 200031, China; ^2^Key Laboratory of Myopia, Ministry of Health, Shanghai Key Laboratory of Visual Impairment and Restoration, Shanghai 200031, China

## Abstract

**Methods:**

A total of 57 cataract patients (57 eyes) with regular corneal astigmatism (≥2.57 D) were enrolled in this retrospective cohort study. Phacoemulsification with toric IOL implantation was performed for all patients. The uncorrected visual acuity (UCVA) and best corrected visual acuity (BCVA) were recorded before and one year after surgery, and statistical analysis of preoperative corneal astigmatism, postoperative residual astigmatism, aberrations, IOL rotation, and related factors was performed to evaluate the efficacy, safety, and stability of toric IOLs in correcting moderate-to-high corneal astigmatism.

**Results:**

One year after surgery, visual acuity was significantly improved compared with that before surgery (preoperative log MAR 0.87 ± 0.34 vs. postoperative log MAR 0.31 ± 0.26, *p* < 0.001), and the self-reported spectacle independence rate was 68.42%. The total residual astigmatism was 1.18 ± 0.85 D, which was significantly less than the preoperative value (3.41 ± 0.99 D) (*p* < 0.001). The degree of toric IOL rotation was 4.93 ± 3.02°, and 54.39% of patients had a lens rotation of less than 5°. The IOLs of 5.26% (3 eyes) of patients rotated more than 10°, and these patients received glasses instead of undergoing IOL repositioning.

**Conclusions:**

Toric IOL implantation provided optimal vision outcomes and low spectacle dependence during a one-year follow-up period. The results from our study show that toric IOL implantation is a safe and effective option for cataract patients with moderate-to-high corneal astigmatism.

## 1. Introduction

Due to recent advances in phacoemulsification and intraocular lenses (IOLs), contemporary cataract surgery has transitioned from rehabilitative surgery to refractive surgery [1]. Safety, accuracy, and predictability are becoming the main indicators in terms of the evaluation of treatment [2]. Astigmatism, one kind of ametropia caused by refractive aberration of the cornea or lens, is very common according to the epidemiological research conducted in China and other countries [3–5]. Approximately 8% to 15% of people without cataracts and 15% to 29% of cataract patients have moderate-to-high corneal astigmatism by more than 1.0 D [6]. Astigmatism not only plays a key role in patients' visual acuity and quality of life but also is a main factor influencing postoperative visual acuity [4]. Currently, the widely used methods to correct corneal astigmatism clinically include spectacles, contact lenses, limbal relaxing incisions (LRIs), arcuate keratotomy, excimer laser astigmatism correction, conductive keratoplasty, and refractive lens exchange (RLE) with the implantation of toric intraocular lenses (toric IOLs) [6–8].

While corneal astigmatism by less than 1.5 D can be corrected by an appropriate surgical incision, the more ideal way to manage moderate-to-high corneal astigmatism is toric IOL implantation [9, 10]. Various studies have demonstrated that toric IOLs can correct low-to-moderate corneal astigmatism with optimal efficacy and stability [11, 12]. However, the long-term clinical outcomes of toric IOL implantation in cataract patients with moderate-to-high corneal astigmatism have not been sufficiently observed.

In this study, we observed the clinical efficacy of toric IOL (SN60T6-T9) implantation in cataract patients with moderate-to-high corneal astigmatism and aimed to evaluate the safety and rotational stability of toric IOLs.

## 2. Patients and Methods

### 2.1. Demographic Characteristics and Medical Histories

The Institutional Review Board (IRB) of the Eye and ENT Hospital of Fudan University approved this study, which adhered to the tenets of the Declaration of Helsinki. Informed consent was obtained from the subjects.

Fifty-seven eyes (from 28 males (49.1%) and 29 females (50.9%); mean age, 46.37 ± 20.20 years, range 12–82 years) that underwent phacoemulsification with the implantation of toric IOLs in the Department of Ophthalmology at the Eye and ENT Hospital of Fudan University between January 2017 and September 2018 were consecutively included. Patients with preoperative corneal astigmatism greater than 2.57 D were eligible for study inclusion if they had no contraindications for toric IOL implantation. Additional eligibility criteria were that patients presented good treatment compliance and had no cognitive or mental disorders. Eyes with irregular astigmatism (defined as the condition when the 2 principal astigmatic meridians of the cornea are not orthogonal) [13] and ocular pathologies other than cataracts (trauma, inflammation, corneal diseases, glaucoma, fundus diseases, Fuchs endothelial dystrophy, tear-film abnormalities (Schirmer ≤ 7 mm/5 min) [14], etc.) were excluded. Patients with a history of ocular surgery and a very small pupil that could not be fully dilated were excluded. All cataract patients were assessed with the Lens Opacity Classification System (LOCS) III classification standard assessment (36 eyes of Nuclear III; 21 eyes of Nuclear IV).

For the analysis of postoperative outcomes, patients were subdivided into groups according to the IOL model implanted as follows: the T6 group (SN60T6), T7 group (SN60T7), T8 group (SN60T8), and T9 group (SN60T9).

### 2.2. Preoperative Assessment

Preoperatively, all patients underwent a standard comprehensive ocular examination. This included a slit lamp examination, fundoscopy assessment, B-scan ultrasonography assessment, axial length measurement (IOL Master 700, Carl Zeiss Meditec, Germany), corneal topography scan (Pentacam HR, OCULUS Optikgeräte, Wetzlar, Germany), corneal endothelial cell count, optical coherence tomography (OCT) scan, measurements of uncorrected visual acuity (UCVA) and best corrected visual acuity (BCVA) with Snellen charts, and Schirmer's test with anesthesia and subjective refraction.

### 2.3. Intraocular Lens

In all patients, the IOL power was calculated using biometry measurements obtained with the IOL master 700 calculated using the 3^rd^- or 4^th^-generation formula (Haigis when the axial length < 22 mm or ≥28 mm; SRK/T when the axial length is from 22 to 28 mm) wherever applicable. The toric IOL model (cylinder power), alignment axis, and anticipated residual astigmatism were calculated using the web-based toric IOL calculator program (Barret) available at http://www.acrysoftoriccalculator.com.

We investigated the following 4 AcrySof toric IOL models in this study: SN60T6, SN60T7, SN60T8, and SN60T9. All toric IOL models are hydrophobic acrylic with a 6.0 mm optic diameter and open-loop-modified L-haptics with stable force haptic design for rotational stability.

The incision location was determined according to the surgeon's preference as follows: temporal incisions were performed by Y. L. and superior incisions were performed by J. Y. The expected amount of surgically induced astigmatism (SIA) based on the surgeon's personal experience varied between 0.35 D and 0.50 D.

### 2.4. Surgical Technique

Cataract surgery was performed by two experienced surgeons (Y. L. and J. Y.). The patients received topical anesthesia. The eyes were dilated to a pupil diameter of at least 8 mm. Intraoperatively, a 2.65 mm clear corneal incision was made according to the surgeon's preference. After a 5–5.5 mm continuous curvilinear capsulorhexis (CCC) was created with the Verion™ Image-Guided System, phacoemulsification was performed (Centurion®, Alcon Laboratories), followed by the irrigation and aspiration of the residual cortex. The toric IOL (Alcon AcrySof Toric IOL, Alcon Laboratories) was then inserted into the capsular bag with a Monarch II injector (Alcon Laboratories). The IOL was rotated to its desired axis by the Verion system. After the residual viscoelastics were removed, the IOL was aligned to its final position, and the incisions were hydrated. All surgeries were performed in a standardized manner without any intraoperative complications, such as posterior capsular rupture.

Postoperatively, 1% prednisolone acetate eye drops (Allergan Pharmaceutical Ireland, Westport, Ireland), 0.5% levofloxaxcin eye drops (Cravit; Santen Pharmaceutical), and 0.1% pranoprofen eye drops (Pranopulin; Senju Pharmaceutical, Osaka, Japan) were used four times per day, and then, the dosage was tapered over three weeks or as clinically indicated.

### 2.5. Follow-Up

Follow-up examinations were performed one year after surgery. These examinations included measurements of UCVA and BCVA using Snellen charts, corneal astigmatism using Pentacam, OPD-Scan III (Nidek, Japan), and subjective refraction (Nidek, Japan). The alignment of the toric IOL and its axis was determined by OPD-Scan III after pupillary dilation. Spectacle usage was patient reported by asking “Do you use spectacles for all distances?” for determination of spectacle independence [15].

### 2.6. Statistical Analysis

Data analysis was performed using SPSS for Windows (version 13.0, SPSS Inc.). Snellen UCVA and BCVA were converted into log MAR values for the statistical calculations [16]. First, normality and homogeneity tests for variance were carried out for each group of data, and then, *χ*^2^ tests, one-way ANOVA, and paired *t* tests were conducted for the data that satisfied a normal distribution and homogeneity of variance, and nonparametric Kruskal–Wallis tests were conducted for those that did not satisfy a normal distribution and homogeneity of variance. Categorical data are presented as percentages (%), and continuous variables are presented as the mean ± SD. Relationships between continuous variables were assessed using Pearson's correlation analysis. A *p* value of <0.05 was considered statistically significant.

## 3. Results

### 3.1. Total

All subjects underwent phacoemulsification on both eyes. Only the right eye of each patient was included in our analyses. Tables 1–3 show the data of all subjects.

### 3.2. Subgroups

All 57 eyes of 57 patients underwent scheduled examinations. The T6 group comprised 29 eyes (50.8%), the T7 group comprised 14 eyes (24.6%), the T8 group comprised 3 eyes (5.3%), and the T9 group comprised 11 eyes (19.3%). The mean follow-up period was 13.73 ± 1.65 months (range 12 to 18 months). The mean degree of preoperative corneal astigmatism was 3.41 ± 0.99D (Table 1).

### 3.3. Visual Outcomes

Tables 1 and 2 show that all the patients' visual acuity was significantly improved after the implantation of toric IOLs compared with that before surgery (pre-UCVA vs. post-UCVA, *p* < 0.001; pre-BCVA vs. post-BCVA, *p* < 0.001).

### 3.4. Astigmatism

**Figure 1:**
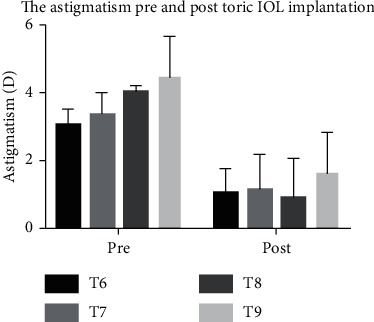
The astigmatism of patients with moderate-to-high corneal astigmatism pre- and posttoric IOL implantation.

There was a statistically significant reduction in astigmatism after toric IOL implantation (Figure 1, Table 2). The postoperative degree of residual astigmatism was 1.18 ± 0.84 D. A significant change in neither the flat nor the steep keratometry values was found after surgery (*p* > 0.05). Spectacle independence for distance vision was reported in 68.42% of the patients with toric IOL implantation. The residual astigmatism was less than 0.75 D in 47.3% of the patients and less than 1.0 D in 54.4% of the patients. The postoperative astigmatism decreased by 65.86% in the T6 group, 66.07% in the T7 group, 77.34% in the T8 group, and 63.57% in the T9 group.

### 3.5. Misalignment and Rotation

The mean decentration of the IOL in total was 0.53 ± 0.33 mm. The mean toric IOL axis rotation was 4.93 ± 3.02° (range, 0 to 15°) at one year (Table 2). The rotation results are shown in Table 3. A total of 54.39% (31 eyes) of patients' IOLs rotated less than 5°. A total of 5.26% (3 eyes) of patients' IOLs rotated more than 10°, and these patients received glasses instead of undergoing IOL repositioning.

### 3.6. Visual Quality

Postoperatively, the total numbers of high-order aberrations (HOAs), spherical aberrations, coma aberrations, and trefoil aberrations were all relatively small, indicating that the patients had better visual quality after the implantation of the toric IOLs (Table 2).

### 3.7. Complications

At the end of the study, 7 eyes had posterior capsule opacification that required neodymium : yttrium-aluminum-garnet (Nd : YAG) laser capsulotomy. In 18 eyes, the postoperative UCVA was worse than 20/40 due to amblyopia (7 eyes), and IOL misalignment (6 eyes) and unexpected residual astigmatism were present (5 eyes).

### 3.8. Correlation Analysis

Postoperative UCVA was correlated with axial length (Pearson's *r* = 0.401, *p*=0.002) and total HOAs (Pearson's *r* = 0.318, *p*=0.016). Postoperative BCVA was correlated with age (Pearson's *r* = 0.410, *p*=0.002) and spherical aberration (Pearson's *r* = 0.296, *p*=0.025). Residual astigmatism was correlated with preoperative astigmatism steep K (Pearson's *r* = −0.319, *p*=0.016), postoperative steep astigmatism K (Pearson's *r* = 0.316, *p*=0.017), total HOAs (Pearson's *r* = 0.385, *p*=0.003), and coma aberrations (Pearson's *r* = 0.477, *p* < 0.001). The decentration distance of the IOL was correlated with the total HOAs (Pearson's *r* = 0.299, *p*=0.024) and coma aberrations (Pearson's *r* = 0.295, *p*=0.026). The degree of rotation was correlated with BCVA (Pearson's *r* = 0.559, *p* < 0.001), residual astigmatism (Pearson's *r* = 0.665, *p* < 0.001), total HOAs (Pearson's *r* = 0.459, *p* < 0.001), and coma aberrations (Pearson's *r* = 0.536, *p* < 0.001). The total HOAs were correlated with axial length (Pearson's *r* = 0.472, *p* < 0.001), postoperative UCVA (Pearson's *r* = 0.318, *p*=0.016), spherical aberrations (Pearson's *r* = 0.626, *p* < 0.001), and coma aberrations (Pearson's *r* = 0.656, *p* < 0.001). Spherical aberrations were associated with axial length (Pearson's *r* = 0.286, *p*=0.031), total HOAs (Pearson's *r* = 0.626, *p* < 0.001), coma aberrations (Pearson's *r* = 0.329, *p*=0.002), and trefoil aberrations (Pearson's *r* = 0.379, *p*=0.004). Trefoil aberrations were correlated with axial length (Pearson's *r* = 0.295, *p*=0.026) and spherical aberrations (Pearson's *r* = 0.379, *p*=0.004).

## 4. Discussion

Cataracts remain the leading cause of blindness worldwide [1]. According to the WHO, there are currently 20 million people with severely reduced vision of 3/60 or worse as a result of cataracts, and this number is expected to be as high as 40 million by 2020 [2, 17]. Currently, due to a lack of safe and effective drug treatment for age-related cataracts, surgery remains the only effective cure [17]. Astigmatism, a common ocular refractive error, affects the postoperative vision of cataract patients who undergo traditional IOL implantation, thus making it difficult for them to achieve postoperative spectacle independence [4, 5]. With the use of toric IOLs, the uncertainty of traditional astigmatism correction surgery and possible corneal complications has been avoided, and the postoperative need for spectacle-free surgery has been met [6]. Toric IOLs can correct preexisting corneal astigmatism in cataract patients, yielding optimal postoperative results under accurate axial alignment [7–13, 18].

AcrySof toric IOLs can correct astigmatism by a cylinder power of up to 6.0 D [8]. Several studies have confirmed that toric IOLs have good efficacy and safety in the correction of low and moderate astigmatism (SN60T3∼SN60T5); therefore, they can effectively improve the visual acuity of patients, with a high off-glass rate and good rotational stability [6–8, 11, 18]. However, relatively few studies have focused on the clinical outcomes of toric IOL implantation in patients with high astigmatism [9, 10, 19–21]. Compared with patients with low and moderate astigmatism, patients with high astigmatism are more affected by residual astigmatism and its predictability [19–21]. This study indicated that toric IOLs can significantly reduce astigmatism in patients with high astigmatism by 63–77%. Consistent with previous studies, the effectiveness of astigmatism correction was shown in our study. We analyzed residual astigmatism by evaluating the residual refractive cylinder in this study. In other studies, changes in astigmatic refraction were also analyzed using vector analysis based on the Alpins method [13].

The poor postoperative visual acuity of some patients was related to amblyopia, refractive error, and IOL rotation. To investigate the long-term efficacy and safety, we analyzed only the one-year follow-up data in our study. Usually, at our hospital, all patients are followed up on the first and third days and at one week, one month, six months, and one year, but not all the patients could finish all follow-ups accordingly. We enrolled only patients who finished the one-year follow-up in this study.

CCC navigated by the VERION™ system was applied during phacoemulsification with toric IOL implantation [22]. A 5.0–5.5 mm capsulorhexis ensured the regularity and centrality of the IOL, which enabled the IOL to be stabilized in the capsular bag. Given that the accuracy of the IOL axial position will greatly affect the postoperative vision of patients with high astigmatism, the intraoperative navigation system used in this study can be used to avoid differences in eye positioning between the sitting and supine positions, as well as errors in manual labeling [7, 22, 23]. Moreover, the adhesion between the biomaterials of the AcrySof toric IOL and the capsule contributes remarkably to maintaining the long-term transparency of the posterior capsule [24, 25], as does the design of the sharp optical edge of the posterior surface, which decreases the posterior capsule opacity (PCO) rate [26]. Compared with previous silicone IOLs, these IOLs had a considerably smaller degree of postoperative IOL rotation [27].

Correct IOL axial placement and IOL rotational stability in the capsule are mandatory for the success of toric IOL implantation since it has been estimated that approximately 1 degree of off-axis IOL rotation leads to a loss of up to 3.3% of IOL cylinder power [28]. Accurate alignment of the toric IOL requires long-term rotational stability after the operation. The investigated toric IOL performed well with regard to improving spectacle independence for distance vision. Astigmatism can be corrected to its greatest extent with an accurate axial position [18]; therefore, the IOL should be placed in the correct axial position to the greatest extent possible during the operation. The IOL will rotate in the capsule shortly after surgery until the capsule shrinks [23]. Hence, the adhesion properties of the IOL enable it to adhere to the capsule immediately after implantation and then maintain its position [24, 25]. Previous studies have shown that good visual outcomes are obtained postoperatively when the degree of toric IOL rotation is within 4° [27, 28]. The residual astigmatism in this study was 1.175 ± 0.841 D, and the rotation was 4.930 ± 3.023°, indicating 17% residual astigmatism in patients with moderate-to-high corneal astigmatism.

Indications should be reasonably selected when toric IOL implantation is used to correct corneal astigmatism in cataract patients. We did not exclude the patients with amblyopia. In another study, toric IOL implantation was also a safe and effective option for congenital cataract patients with corneal astigmatism. Visual impairment in congenital cataract patients may be caused by dense central opacity of the lens or high degrees of astigmatism. To fully evaluate the efficacy and safety of toric IOL implantation in patients with moderate-to-high corneal astigmatism, we also enrolled patients who may have had amblyopia. Patients with Fuchs' endothelial corneal dystrophy (FECD) can undergo corneal transplantation in the future; therefore, toric IOL implantation is still challenging [29]. Toric IOL implantation is most suitable for patients with “bow-tie”-shaped regular astigmatism [6]. Therefore, it is crucial to determine the type of corneal astigmatism by corneal topography before surgery. Recently, a number of studies have evaluated the effects of toric IOL implantation on patients with irregular corneal astigmatism, such as corneal transplantation or radial keratotomy, suggesting that toric IOL implantation can partially correct astigmatism [30–33].

## 5. Conclusions

The investigated toric IOLs performed well, as they improved the uncorrected distance visual acuity, reduced corneal astigmatism, and yielded spectacle independence in cataract patients with moderate-to-high astigmatism while presenting potentially good rotational stability and predictability. Thus, these lenses are safe and effective for the long-term correction of preoperative moderate-to-high corneal astigmatism. Additional prospective comparative studies with larger sample sizes and longer follow-up periods should be conducted to further refine the applications of toric IOL implantation in patients with moderate-to-high corneal astigmatism.

## Figures and Tables

**Table 1:** Preoperative patient demographic data.

	Subjects (eye)	Sex (M/F)	Mean age (years)	UCVA (log MAR)	BCVA (log MAR)	Average corneal cylinder (preoperation) (D)	Flat *K* (D)	Steep *K* (D)	Mean axial length (mm)
T6	29	9/20	48.79 ± 18.44	0.85 ± 0.40	0.80 ± 0.41	3.06 ± 0.48	42.46 ± 1.84	45.51 ± 1.66	25.33 ± 2.37
T7	14	8/6	44.86 ± 22.90	0.75 ± 0.30	0.69 ± 0.31	3.37 ± 0.65	41.85 ± 1.35	45.22 ± 1.56	24.65 ± 2.23
T8	3	2/1	45.33 ± 32.53	1.10 ± 0.17	0.63 ± 0.08	4.05 ± 0.17	41.94 ± 2.37	45.99 ± 2.54	24.67 ± 1.34
T9	11	9/2	42.18 ± 20.00	1.00 ± 0.18	0.56 ± 0.10	4.49 ± 1.19	42.00 ± 1.57	46.49 ± 1.74	24.50 ± 1.80
Total	57	28/29	46.37 ± 20.20	0.87 ± 0.34	0.72 ± 0.35	3.41 ± 0.99	42.19 ± 1.67	45.66 ± 1.72	24.97 ± 2.18

UCVA, uncorrected visual acuity; BCVA, best corrected visual acuity.

**Table 2:** Outcome analysis after cataract surgery with a high-power toric intraocular lens.

	UCVA (logMAR)	BCVA (logMAR)	Residual astigmatism (D)	Flat *K* (D)	Steep *K* (D)	IOL rotation (degree)	Decentration (mm)	Total internal HOAs	Spherical aberrations	Coma aberrations	Trefoil aberrations
**T6**	0.36 ± 0.30	0.10 ± 0.14	1.04 ± 0.73	42.67 ± 1.72	45.33 ± 1.82	5.24 ± 3.10	0.50 ± 0.23	0.87 ± 0.62	0.02 ± 0.04	0.05 ± 0.05	0.19 ± 0.24
**T7**	0.22 ± 0.15	0.07 ± 0.10	1.14 ± 1.06	42.02 ± 1.34	45.26 ± 1.66	4.79 ± 3.91	0.61 ± 0.52	0.73 ± 0.55	0.01 ± 0.01	0.06 ± 0.11	0.22 ± 0.37
**T8**	0.17 ± 0.06	0.07 ± 0.06	0.92 ± 1.16	43.22 ± 1.60	46.65 ± 1.54	4.67 ± 2.08	0.68 ± 0.08	0.72 ± 0.56	0.01 ± 0.01	0.10 ± 0.11	0.09 ± 0.06
**T9**	0.35 ± 0.29	0.05 ± 0.09	1.64 ± 1.21	42.29 ± 1.43	46.73 ± 1.75	4.36 ± 1.69	0.46 ± 0.27	0.74 ± 0.24	0.02 ± 0.02	0.05 ± 0.04	0.14 ± 0.09
**Total**	0.31 ± 0.26	0.08 ± 0.12	1.18 ± 0.84	42.47 ± 1.57	45.65 ± 1.82	4.93 ± 3.02	0.53 ± 0.33	0.80 ± 0.54	0.02 ± 0.03	0.06 ± 0.07	0.18 ± 0.25

UCVA, uncorrected visual acuity; BCVA, best corrected visual acuity; HOA, high-order aberration.

**Table 3:** Rotation after cataract surgery with a high-power toric intraocular lens.

Rotation (degree)	Subjects	Percentage
0	1	1.8
1	2	3.5
2	7	12.3
3	10	17.5
4	11	19.3
5	7	12.3
6	6	10.5
7	4	7.0
8	5	8.8
10	1	1.8
12	1	1.8
15	2	3.5

## Data Availability

The clinical data used to support the findings of this study were provided by the Eye and ENT Hospital of Fudan University under license; therefore, they cannot be made freely available. Access to these data will be considered by the author upon request, with permission from the Eye and ENT Hospital of Fudan University.

## Additional Points

Synopsis/precis: in cataract patients with moderate-to-high corneal astigmatism, toric IOL implantation is a safe and effective option and provides optimal vision outcomes with good rotational stability and low spectacle dependence over a one-year follow-up period.
